# Unveiling behavioral and molecular neuroadaptations related to the antidepressant action of cannabidiol in the unpredictable chronic mild stress model

**DOI:** 10.3389/fphar.2023.1171646

**Published:** 2023-04-18

**Authors:** María Salud García-Gutiérrez, Daniela Navarro, Amaya Austrich-Olivares, Jorge Manzanares

**Affiliations:** ^1^ Instituto de Neurociencias, Universidad Miguel Hernández, Alicante, Spain; ^2^ Research Network on Primary Addictions, Instituto de Salud Carlos III, MICINN and FEDER, Madrid, Spain; ^3^ Instituto de Investigación Sanitaria y Biomédica de Alicante (ISABIAL), Alicante, Spain

**Keywords:** cannabidiol, unpredictable chronic mild stress model, sertraline, neuroplasticity, mice, antidepressant properties

## Abstract

**Introduction:** This study aims to further characterize cannabidiol’s pharmacological and molecular profile as an antidepressant.

**Methods:** Effects of cannabidiol (CBD), alone or combined with sertraline (STR), were evaluated in male CD1 mice (*n* = 48) exposed to an unpredictable chronic mild stress (UCMS) procedure. Once the model was established (4 weeks), mice received CBD (20 mg·kg-1, i.p.), STR (10 mg·kg-1, p.o.) or its combination for 28 days. The efficacy of CBD was evaluated using the light-dark box (LDB), elevated plus maze (EPM), tail suspension (TS), sucrose consumption (SC) and novel object recognition (NOR) tests. Gene expression changes in the serotonin transporter, 5-HT1A and 5-HT2A receptors, BDNF, VGlut1 and PPARdelta, were evaluated in the dorsal raphe, hippocampus (Hipp) and amygdala by real-time PCR. Besides, BDNF, NeuN and caspase-3 immunoreactivity were assessed in the Hipp.

**Results:** CBD exerted anxiolytic and antidepressant-like effects at 4 and 7 days of treatment in the LDB and TS tests, respectively. In contrast, STR required 14 days of treatment to show efficacy. CBD improved cognitive impairment and anhedonia more significantly than STR. CBD plus STR showed a similar effect than CBD in the LBD, TST and EPM. However, a worse outcome was observed in the NOR and SI tests. CBD modulates all molecular disturbances induced by UCMS, whereas STR and the combination could not restore 5-HT1A, BDNF and PPARdelta in the Hipp.

**Discussion:** These results pointed out CBD as a potential new antidepressant with faster action and efficiency than STR. Particular attention should be given to the combination of CBD with current SSRI since it appears to produce a negative impact on treatment.

## 1 Introduction

Major depressive disorder (MDD) is one of the most frequently diagnosed mental diseases, affecting more than 260 million people worldwide. According to World Health Organization, this psychiatric disorder presents devastating prevalence, mortality, morbidity, and disability rates (WHO, 2021). With a lifetime prevalence of up to 17%, MDD is the leading cause of disability worldwide and the fourth leading cause of disease burden (Friedrich, 2017; WHO, 2021). Suffering MDD reduces average life expectancy by 10–12 years (Jia et al., 2015). Its high comorbidity with other psychiatric diseases, such as anxiety disorders, is one of the main factors contributing to the increased mortality and disability associated with this disorder (Ploski and Vaidya, 2021; Serra-Blasco et al., 2021; Bassett et al., 2022; Chen, 2022). The complex symptomatology of this disease, often chronic or recurrent, limits patients’ functioning, affecting their social relationships, including family breakdown, absence from work, and reduced productivity in the workplace. Likewise, depressive disorders represent one of the main risk factors for suicide (Hawton et al., 2013).

Despite the significant impact of MDD, the biological bases of this disease remain incompletely understood. This lack of knowledge is the main reason for the low clinical response rate of current antidepressant drugs. Indeed, only one-third of patients achieve complete remission. A series of trials sponsored by the National Institute of Mental Health (NIMH) in the United States provided relevant data. In the Sequenced Treatment Alternatives to Relieve Depression (STAR*D) study, only 31% of patients with MDD were in remission after treatment with a selective serotonin reuptake inhibitor for 14 weeks (Fava et al., 2003; Rush et al., 2004; Rush et al., 2006). All these results highlight the urgent need to identify new drugs with a different mechanism of action than classical antidepressants that improve the clinical outcome and our understanding of the molecular mechanisms of depression.

Cannabidiol (CBD), one of the main compounds in the plant Cannabis sativa, presents a wide range of pharmacological properties, such as anxiolytic, antidepressant, antipsychotic, anticonvulsant and neuroprotective (Garcia-Gutierrez et al., 2020; Kwee et al., 2022; Atalay Ekiner et al., 2022; Yousaf et al., 2022; V Micale et al., 2015; Stark et al., 2021; Di Bartolomeo et al., 2021; Stark et al., 2019). These actions have considerably increased the number of studies aimed at clarifying its therapeutic role in various neuropsychiatric diseases, including depressive disorders. Besides, CBD does not display potential as a drug of abuse according to animal models (Parker et al., 2004; Vann et al., 2008; Viudez-Martinez et al., 2019) and open trials carried out with healthy volunteers (Fusar-Poli et al., 2009; Winton-Brown et al., 2011; Martin-Santos et al., 2012).

Cumulative evidence showed that CBD reduces anxiety and behavioral despair in rats and mice, depending on the dose and the strain of rodents evaluated (Guimaraes et al., 1990; Moreira et al., 2006; Resstel et al., 2006; Casarotto et al., 2010; Reus et al., 2011; Viudez-Martinez et al., 2018). However, most of these studies used acute behavioral tests with little power of clinical translation because they partially reproduce the clinical condition (Krishnan and Nestler, 2008). In this respect, the unpredictable chronic mild stress model (UCMS) is a well-validated animal model of depression due to its reliable predictive face and construct validities. It is the ideal model for selecting optimal antidepressants (Willner, 1997; Willner, 2017; Nollet, 2021). To date, only a few studies have evaluated CBD effects using this animal model. The results reported by these studies indicate that both acutely and chronically CBD administration reduces depressive-like behaviors, including anhedonia. Although these results appear promising, they also present certain limitations. Gall et al. (2020) administered CBD from the beginning of the UCMS, hampering the possibility of evaluating its ability to modulate depressive-like behaviors once already established. In the study by Xu et al. (2019), CBD was administered weekly for 2 weeks, starting the treatment from the second week of the UCMS, evaluating CBD effects only on the forced swimming test. Finally, Ma et al. (2021) only assessed the impact of an acute administration of CBD, remaining to be demonstrated its sustained and long-term effects of a chronic administration. More importantly, CBD has not been compared to any reference antidepressant drug.

This study aims to answer fundamental aspects necessary to further characterize CBD’s pharmacological and molecular profile as an antidepressant. Thus, we evaluated CBD antidepressant-like effects in mice exposed to the UCMS. The treatment started on the 4-week of the UCMS, once the model was established, and was given for 28 days. Antidepressant-like effects were assessed by using a complete behavioral test battery evaluating anxiety (light-dark box and elevated plus maze), behavioral despair (tail suspension test), cognitive impairment (novel object recognition) and anhedonia (sucrose intake). Furthermore, we compared if CBD displays antidepressant-like effects faster than sertraline (STR), an antidepressant reference drug, and/or its effectiveness in modulating the behavioral and molecular disturbances induced by the UCMS was different. In addition, we analyzed if the combination of CBD and STR may present additive or synergistic effects. The molecular neuroadaptations closely related to its mechanism of action were analyzed by measuring changes in the gene expression of key targets of the serotoninergic system (serotonin transporter reuptake (Scl6a4), 5-HT1A and 5-HT2A serotonin receptors), neuroplasticity (BDNF, Vglut1, PPARdelta) in the dorsal raphe (DR), hippocampus (Hipp), and amygdala (AMY) of stressed mice by real-time PCR. Immunohistological studies also evaluated BDNF, NeuN and caspase-3 immunoreactivity in the Hipp.

## 2 Materials and methods

### 2.1 Animals

Male CD1 mice were used in all experiments (Charles River, Barcelona, Spain). At the beginning of the experiments, mice were 5 weeks old and weighed 20–25 g. All animals were maintained under a controlled temperature (23°C ± 2°C) and with a light-dark cycle from 08.00 to 20.00 h, with free access to food (commercial diet for rodents A04 Panlab, Barcelona, Spain) and water. All the behavioral tests were carried out between 8.00 and 14.00 h. All the studies were conducted in compliance with the Spanish Royal Decree 1201/2005, the Spanish Law 32/2007, and the European Union Directive of 22 September 2010 (2010/63/UE), regulating the care of experimental animals.

### 2.2 Treatments

CBD was obtained from STI Pharmaceuticals (Essex, United Kingdom), dissolved in ethanol:cremophor:saline (1:1:18) and injected i.p. at the correspondent dose (20 mg·kg-1 in 0.3 mL of solution, twice daily). This dose was selected based on our previous dose-response experiments in CD1-stressed mice exposed to the UCMS (Supplemenary Figure S1).

STR was purchased (Besitran, Pfizer, 20 mg·ml-1, concentrated oral solution), dissolved in water and administered p.o. at the corresponding dose (10 mg·kg-1 in 0.3 mL of solution, once daily). This dose was selected based on previous studies (Lu et al., 2019; Zhang et al., 2022).

For mice receiving the combination of CBD and STR, drugs were prepared as described above and given CBD + STR during the morning and CBD + VEH during the evening.

### 2.3 Behavioral analyses

#### 2.3.1 Chronic unpredictable mild stress

Mice were exposed to UCMS for 8 weeks, following the previously published protocol with some modifications (Willner et al., 1992; Willner, 1997; Garcia-Gutierrez et al., 2010). Briefly, mice were exposed several times a day to one or more of the following stressful stimuli (stressors): wet cage, food deprivation, restraint stress, period of stroboscopic illumination (150 flashes·min-1), inversion of light/dark cycle, tilted cage (45°), loud noise (90–105 dB) and fox urine exposure. All stressors and/or sequences were applied at different time points to avoid habituation and add unpredictability to the stressors (Table 1).

**TABLE 1:** The chronic unpredictable mild stress procedure.

	Monday	Tuesday	Wednesday	Thursday	Friday	Saturday	Sunday
Week 1	10–11 h Restraint Stress	9–12 h Stroboscopic illumination	10–10.30 h Fox urine exposure	10–13 h Stroboscopic illumination	9.00–9.30 h Fox urine exposure	8.30–19 h Inversion light/dark cycle	10–11 h Retraint Stress
	12–15 h Stroboscopic illumination	13–14 h Restraint Stress	15–17 h Loud noise	14–15 h Restraint Stress	13–14 h Restraint Stress		19-8.30 h (+1 day)
	17–18 h Loud noise	19-8 h (+1 day) Titled cage	18–19 h Restraint Stress	16–18 h Loud noise	16–19 h Stroboscopic illumination		Wet cage
Week 2	11–13 h Stroboscopic illumination	10–11 h Restraint Stress	**8-14h Behavioral test= LDB**	9–11 h Loud noise	10–13 h Stroboscopic illumination	10.30–12.30 h Loud noise	8.30h–14 h Inversion light/dark cycle
	16–18 h Loud noise	13–13.30 h Fox urine exposure	18–19 h Restraint stress	12–15 h Stroboscopic illumination	19-8.30 h (+1 day) Tilted cage	15–15.30 h Fox urine exposure	16–17 h Restraint stress
		16–18 h Loud noise		17–17.30 h Fox urine exposure			
Week 3	10–11 h Restraint Stress	**8-14h Behavioral test= EPM**	9–11 h Loud noise	10–13 h Stroboscopic illumination	10–10.30 h Fox urine exposure	9–18 h Wet cage	8.30–19 h Inversion light/dark cycle
	12–14 h Loud noise	18–18.30 h Fox urine exposure	12–15 h Stroboscopic illumination	19-8.30 h (+1 day) Tilted cage	13–14 h Restraint Stress		
	16–18 h Stroboscopic illumination		18–19 h Restraint Stress		16–19 h Stroboscopic illumination		
Week 4	**8-14h Behavioral test= TS**	10–11 h Restraint Stress	9–11 h Loud noise	9–12 h Stroboscopic illumination	11–11.30 h Fox urine exposure	19-13 h (+1 day) Food and water deprivation	**13-14h Behavioral test= SI**
	17–19 h Loud noise	13–13.30 h Fox urine exposure	12–15 h Stroboscopic illumination	13–14 h Restraint Stress	13–14 h Restraint Stress		
		16–19 h Stroboscopic illumination	18–19 h Restraint stress	19–8.00 (+1 day) Wet cage	19-14 h (+1 day) Titled cage		
Week 5	**Start of treatment**	9–11 h Loud noise	10–13 h Stroboscopic illumination	**8-14h Behavioral test= LDB**	9–11 h Loud noise	8.30–19 h Inversion light/dark cycle	**8.00-14h Behavioral test= TS**
	10–11 h Restraint Stress	13–14 h Restraint Stress	15–17 h Loud noise	18–18.30 h Fox urine exposure	12–15 h Stroboscopic illumination		
	13–13.30 h Fox urine exposure	19-8 h (+1 day) Titled cage	18–19 h Retraint stress		18–19 h Restraint stress		
Week 6	11–13 h Stroboscopic illumination	10–11 h Restraint Stress	10–12 h Loud noise	10–13 h Stroboscopic illumination	11–13 h Loud noise	9–10 h Restraint Stress	**8.00-14h Behavioral test= EPM**
	16–18 h Loud noise	13–15 h Loud noise	13–13.30 h Fox urine exposure	14–15 h Restraint Stress	16–19 h Stroboscopic illumination	12–19 h Tilted cage	
		16–19 h Stroboscopic illumination	16–17 h Restraint Stress	19-8 h (+1 day) Wet cage			
Week 7	9–12 h Stroboscopic illumination	10–11 h Restraint Stress	**8-14h Behavioral test= NOR** 16–18 h Loud noise	**8-14h Behavioral test= NOR** 17–18 h Restraint Stress	10–10.30 h Fox urine exposure	9.30–11.30 h Loud noise	8.30–19 h Inversion light/dark cycle
	13–14 h Restraint Stress	12–14 h Loud noise			12–15 h Stroboscopic illumination	14–19 h Wet cage	
	19-8 h (+1 day) Titled cage	16–18 h Stroboscopic illumination			18–19 h Restraint stress		
Week 8	9–11 h Loud noise	10–13 h Stroboscopic illumination	10–11 h Restraint Stress	11–11.30 h Fox urine exposure	**13-14h Behavioral test= SI**		
	13–14 h Restraint Stress	15–17 h Loud noise	12–15 h Stroboscopic illumination	13–14 h Restraint stress			
	19-8 h (+1 day) Titled cage	18–19 h Restraint stress	17–19 h Loud noise	19-13 h (+1 day) Food and water deprivation			

LBD: light-dark box; EPM: elevated plus maze; TS: tail suspension; NOR: novel object recognition; SI: sucrose intake. Regarding bold values, are the hours at which mice were exposed to the stimuli or the schedule in which the behavioral tests were performed.

**FIGURE 1:**
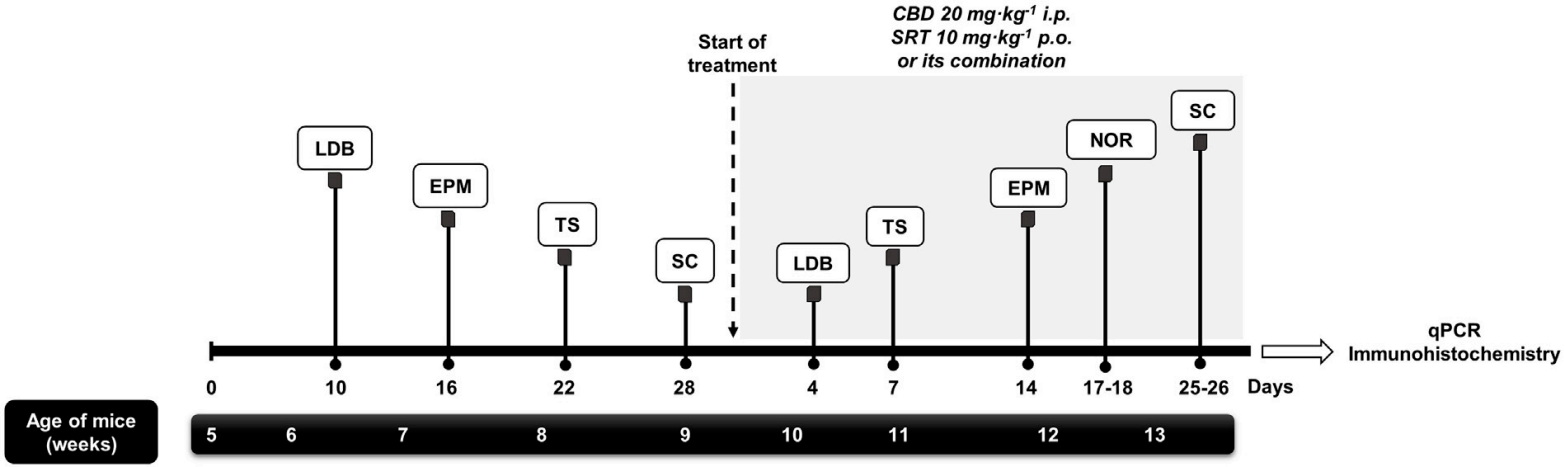
**Schematic representation of the experimental design**. Mice were exposed to the Unpredictable Chronic Mild Stress Model (UCMS) for 8 weeks. During the first 4 weeks, the model was established, being behavioral alterations evaluated at 10, 16, 22 and 28 days by the light-dark box (LDB), elevated plus maze (EPM), tail suspension (TS) and sucrose consumption (SC) tests, respectively. Once the model was established, mice were distributed randomly to receive CBD (20 mg·kg-1, i.p., twice daily, at 9.00h and 19.00 h), STR (10 mg·kg-1, p.o., once daily, at 9.00 h), their combination or VEH, during 28 days. The efficacy of each treatment in modulating the effects of UCMS were evaluated at different time points by the LBD, TS, EPM, Novel Object Recognition (NOR) and SC tests. Finally, mice were decapitated or perfused to perform real-time PCR (qPCR) and immunohistological studies.

During the exposure to the UCMS, behavioral alterations were analyzed using different tests at various time points (Figure 1). Once the UCMS was established (4 weeks), drug treatment was initiated, randomly dividing the mice into the following groups: 1) VEH-treated UCMS, 2) CBD-treated UCMS, 3) CBD + STR-treated UCMS, 4) STR-treated UCMS. Mice not exposed to the UCMS were also treated with the VEH of CBD and the VEH of STR (VEH-treated non-UCMS). Treatment was given for a total of 28 days.

#### 2.3.2 Light-dark box test (LDB)

This test uses the natural aversion of rodents to bright areas compared with darker ones (Crawley and Goodwin, 1980; Garcia-Gutierrez and Manzanares, 2011). In a two-compartment box, rodents prefer dark areas, whereas anxiolytic drugs should increase the time spent in the light compartment. The apparatus consisted of two methacrylate boxes (20 × 20 × 15 cm), one transparent and one black and opaque, separated by an opaque tunnel (4 cm). Light from 60 W desk lamp placed 25 cm above the light box provided room illumination. Mice were individually tested in 5 min sessions. During this period, the time spent in the light box and the number of transitions between the two compartments were recorded. A mouse whose four paws were in the new box was considered as having changed boxes. The floor of each box was cleaned between sessions with paper towels moistened with ethanol 70% and thoroughly dried. At the beginning of the session, mice were placed in the lightbox facing the tunnel that connects to the dark box.

#### 2.3.3 Elevated plus maze (EPM)

This test consisted of two open arms and two enclosed horizontal perpendicular arms 50 cm above the floor (Lister, 1987; Garcia-Gutierrez and Manzanares, 2011). The junction of four arms formed a central squared platform (5 × 5 cm). The test began with the animal being placed in the center of the apparatus facing one of the enclosed arms and allowed to explore freely for 5 min. During this period, the time spent in the open arms (as percentages of total test time) and the number of entries from open arms to closed arms (and *vice versa*) were recorded. An arm entry was considered the entry of four paws into the arm. The floor of each arm was cleaned between sessions with paper towels moistened with ethanol 70% and fully dried.

#### 2.3.4 Tail suspension test (TS)

Mice were individually suspended by the tail at the edge of a lever above the table top (the distance to the table surface was 35 cm), affixed with the adhesive tape placed approximately 1–2 cm from the tip of the tail (Vaugeois et al., 1997; Garcia-Gutierrez et al., 2010). The duration of immobility was measured for 6 min. In this situation, mice develop escape-orientated behaviors interspersed with temporally increasing bouts of immobility.

#### 2.3.5 Novel object recognition test (NOR)

Rodents have an innate preference towards novelty, meaning less cognitive impairment. This paradigm is based on the natural tendency of mice to explore new objects and environments and compare them with familiar ones (Busquets-Garcia et al., 2018; Brambilla-Pisoni et al., 2022). The NOR was carried out in an open field cage of 40 × 40 × 50 cm of transparent methacrylate with two identical objects in texture, color, size, and shape: Object A (familiar) on the habituation day. On the trial day, one of Objects A remained, and the other was changed with a new different object in texture, color, size and shape: Object B (novel). On the first day, mice were habituated to the arena with two identical Objects A for 5 min. To long-term assessed memory, 24 h later, the habituation took place, mice were exposed to one Object A (familiar object) and Object B (novel object), again for 5 min. In both sessions, the exploration time for each object was quantified. Exploration time was defined as when the animal orientates the nose, sniffs, or touches the object with its front legs at a less or equal distance to 1 cm. The discrimination index was calculated as the difference between time spent exploring the novel and familiar object divided by the total exploration time of the two objects: [(Object B–Object A)/(Object A+ Object B)] (or see formula). Values for the discrimination ratio ranged from 0 to 1, where a score closer to 0 indicated a preference for the familiar object, while a score closer to 1 indicated a greater preference for the novel object. This way, high discrimination index reflects better memory retention for the familiar object.

$$DI=\left(\left(Object\;B-Object\;A\right)/\left(Object\;A+Object\;B\right)\right)$$

#### 2.3.6 Sucrose intake test (SI)

Sucrose intake (5% sucrose solution) was measured after 18 h of food and water deprivation during a period of 1 h (Li et al., 2007). Consumption of sucrose solution was estimated simultaneously in the control and experimental groups by measuring and comparing the volume before and after the 1-h window. Sucrose intake was expressed as mg sucrose·g-1 body weight.

### 2.4 Gene expression studies by real-time PCR

Mice were killed 2 h and a half after the last administration of CBD, STR, its combination or vehicle. Briefly, brains were removed from the skull, frozen over dry ice, and stored at −80°C until the day of the assay. Brain sections of 500 μm were cut at different levels containing the regions of interest (AMY, Hipp and DR), according to Paxinos and Franklin, (2001). Sections were mounted on slides and stored at −80°C. One section of each level was dissected following the method described by Palkovits, (1983). Total RNA was obtained from brain punches using Biozol^®^ Total RNA extraction reagent (Bioflux, Inilab, Madrid, Spain) in the AMY, Hipp and DR areas. After DNAse digestion, reverse transcription was carried out to obtain the complementary DNA (cDNA) following the manufacturer’s instructions (High-Capacity cDNA Reverse Transcription Kit with RNase Inhibitor, Applied Biosystems, Madrid, Spain). Quantitative analyses of the relative gene expression of Scl6a4 (Mm00439391_m1), 5-HT1A (Mm00434106_s1), 5-HT2A (Mm00555764_m1), BDNF (Mm00432069_m1), PPARdelta (Mm00803184_m1), and mVglut1 (Mm00812886_m1) were measured using Taqman Gene Expression assay (Applied Biosystems, Madrid, Spain) as a double-stranded DNA-specific fluorescent dye and performed on the StepOnePlus™ Real-Time PCR System (Applied Biosystems, Madrid, Spain). The reference gene was 18S rRNA, detected using Taqman ribosomal RNA control reagents. Briefly, the data for each target gene were normalized to the endogenous reference gene, and the fold change in target gene abundance was determined using the 2^−ΔΔCT^ method (Livak and Shmittgen, 2001; Garcia-Gutierrez and Manzanares, 2011).

### 2.5 Conventional histology and immunohistochemistry

The general criteria reported by Amaral et al. (1995) were used to define the hippocampal areas and strata. The study focused on the dentate gyrus (DG) and the CA3 and CA1 regions of Cornu Ammonis (CA). Briefly, after 12–14 h of the last administration of the corresponding drug and/or exposure to stressful stimuli, mice were weighed, anesthetized with isoflurane, and intracardially perfused with 50 mL of saline followed by 200 mL of 4% paraformaldehyde, 0.1M sucrose, and 0.002% CaCl2 in 0.1M phosphate buffer (PB; 1.4% K2HPO4 14 g/L, NaH2PO4.2H2O ∼3 g/L to pH 7.3–7.4). Brains were dissected, post-fixed by immersion in the same perfusion medium at room temperature for 4 h and then stored in 0.05% sodium azide in PB at 4°C. Eight parallel series of coronal sections containing the DG and CA rostromedial portion were cut with a Microm HM 650 V vibratome (Thermo Fisher Scientific, Inc., Barcelona, Spain) at 50 μm and stored in 0.05% sodium azide in PB at 4°C. For BDNF, one series was immunostained with rabbit anti-BDNF polyclonal antibody (1:400; EMO, Millipore, United States). All sections were then incubated with goat anti-rabbit antibody, Alexa Fluor 488, labeled (1:200, Molecular Probes, Invitrogen, Barcelona, Spain) and mounted using ProLong Gold (Molecular Probes, Invitrogen).

The adjacent series were double immunostained for fluorescence, starting with mouse anti-neurons neuronal nuclei (NeuN) monoclonal Ab (mAb) (1:400; Chemicon International Inc., Temecula, CA) and rabbit anti-Caspase 3 (1: 300, Cell Signaling Technology, United States). All sections were then incubated with goat anti-mouse antibody, Alexa Fluor Rhodamine Red labeled (1:200, Molecular Probes, Invitrogen, Barcelona, Spain), followed by goat anti-rabbit Alexa Fluor 488 labeled (1:200, Molecular Probes, Invitrogen, Barcelona, Spain).

All samples were examined in a Leica SPEII confocal laser fluorescence microscope, with images captured using Leica Application Suite X software.

### 2.6 Statistical analyses

Statistical analyses were performed using the Student’s t-test for comparing two groups, and one-way analysis of variance (ANOVA) followed by the Student-Newman-Keuls posthoc test for comparing four groups of stressed mice treated with VEH, CBD, STR or its combination. Differences were considered significant if the error probability was less than 5%. SigmaPlot 11 software (Systat Software Inc., Chicago, IL, United States) was used to analyze the data and create the graphs.

## 3 Results

### 3.1 Assessment of behavioral alterations in mice exposed to the UCMS before pharmacological administration

In UCMS, anxiogenic- and depressogenic-like behaviors were evaluated at different time points using the LDB (10 days) and EPM (16 days), and the TS (22 days) and sucrose intake tests (28 days), respectively.

#### 3.1.1 LBD and EPM tests

**FIGURE 2:**
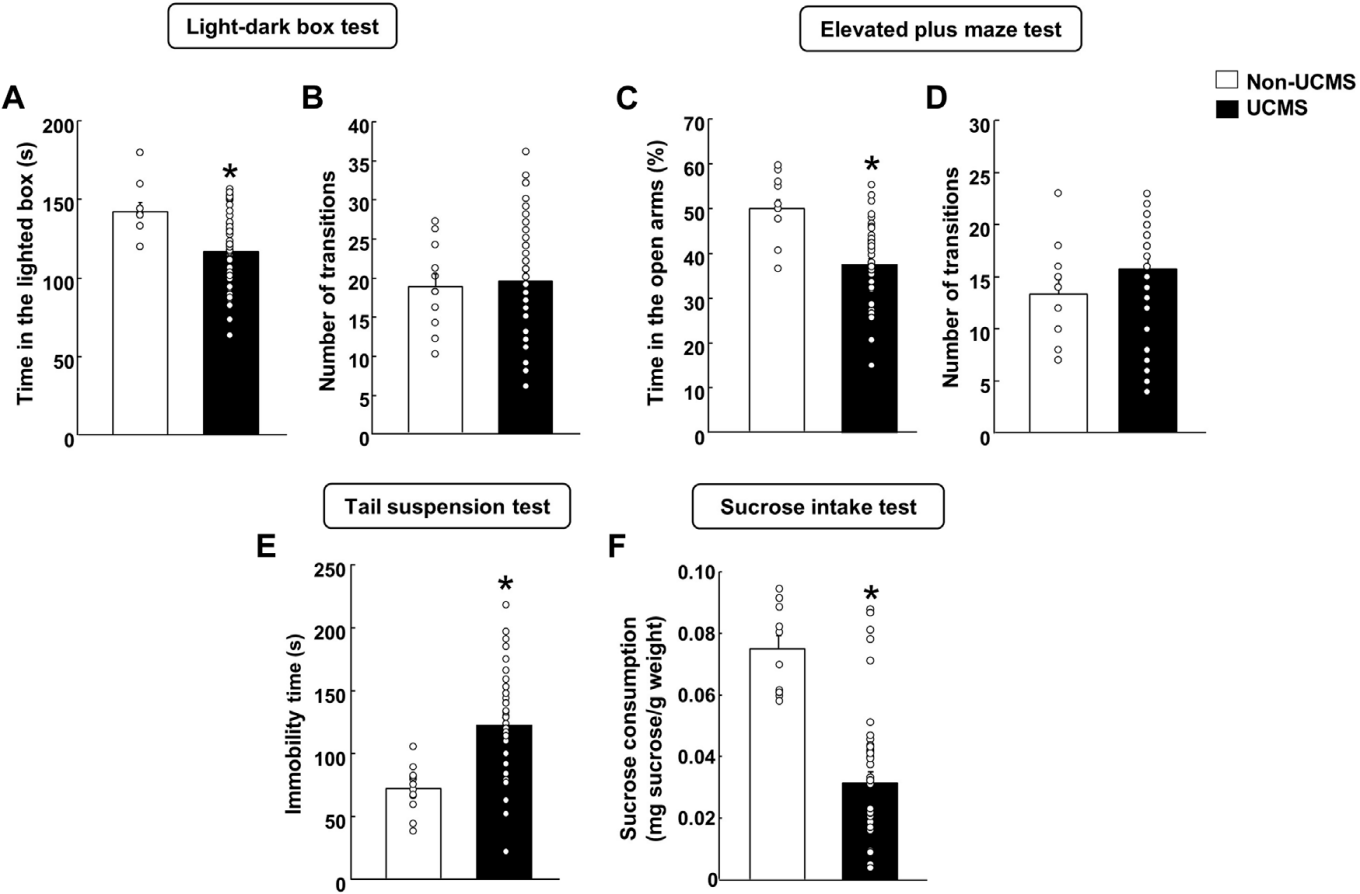
**Behavioral evaluation of mice exposed to the unpredictable chronic mild stress model (UCMS) before starting pharmacological treatment**. Panels **(A, B)** Light-dark box, Panels **(C, D)** Elevated plus maze, Panel **(E)** Tail suspension and Panel **(F)** Sucrose intake. Columns represent the means and vertical lines the ±SEM of each parameter evaluated. *Values from the UCMS-exposed group that differ significantly from the non-UCMS group (Student’s t-test, *p* < 0.05).

In the LDB, stressed mice spent less time in the lighted box compared to the non-UCMS group (Figure 2A) (Student’s t-test: t = 3.641, 57 df, *p* < 0.001) (n = 12 non-UCMS; n = 48 UCMS). No difference was found in the number of transitions (Figure 2B) (Student’s t-test: t = −0.352, 57df, *p* = 0.726) (n = 12 non-UCMS; *n* = 48 UCMS).

Similar results were obtained in the EPM since mice exposed to the UCMS showed a significant reduction in the time spent (%) in the open arms in comparison with the non-UCMS group (Figure 2C) (Student’s t-test: t = 4.873, 57df, *p* < 0.001) (n = 12 non-UCMS; n = 48 UCMS). No difference was observed in the number of transitions (Figure 2D) (Student’s t-test: t = −1.279, 57df, *p* = 0.206) (n = 12 non-UCMS; n = 48 UCMS).

#### 3.1.2 TS test

On day 22, a significant increase in the immobility time was found in mice exposed to the UCMS compared with its corresponding control group (Figure 2E) (Student’s t-test: t = −4.587, 57 df, *p* < 0.001) (n = 12 non-UCMS; *n* = 48 UCMS).

#### 3.1.3 Sucrose intake test

On day 28, stressed mice displayed a significant reduction of sucrose intake (mg sucrose·g-1 body weight) compared with the non-UCMS group (Figure 2F) (Student’s t-test: t = 6.051, 57 df, *p* < 0.001) (n = 12 non-UCMS; n = 48 UCMS).

### 3.2 Effects of chronic CBD, STR and its combination administration on modulating behavioral alterations induced by the UCMS

#### 3.2.1 Effects at 4 days of treatment: LDB test

Firstly, we evaluated if there was a difference in the speed with which the antidepressant-like effect was established between CBD and the reference drug STR using the LBD after 4 days of treatment.

**FIGURE 3:**
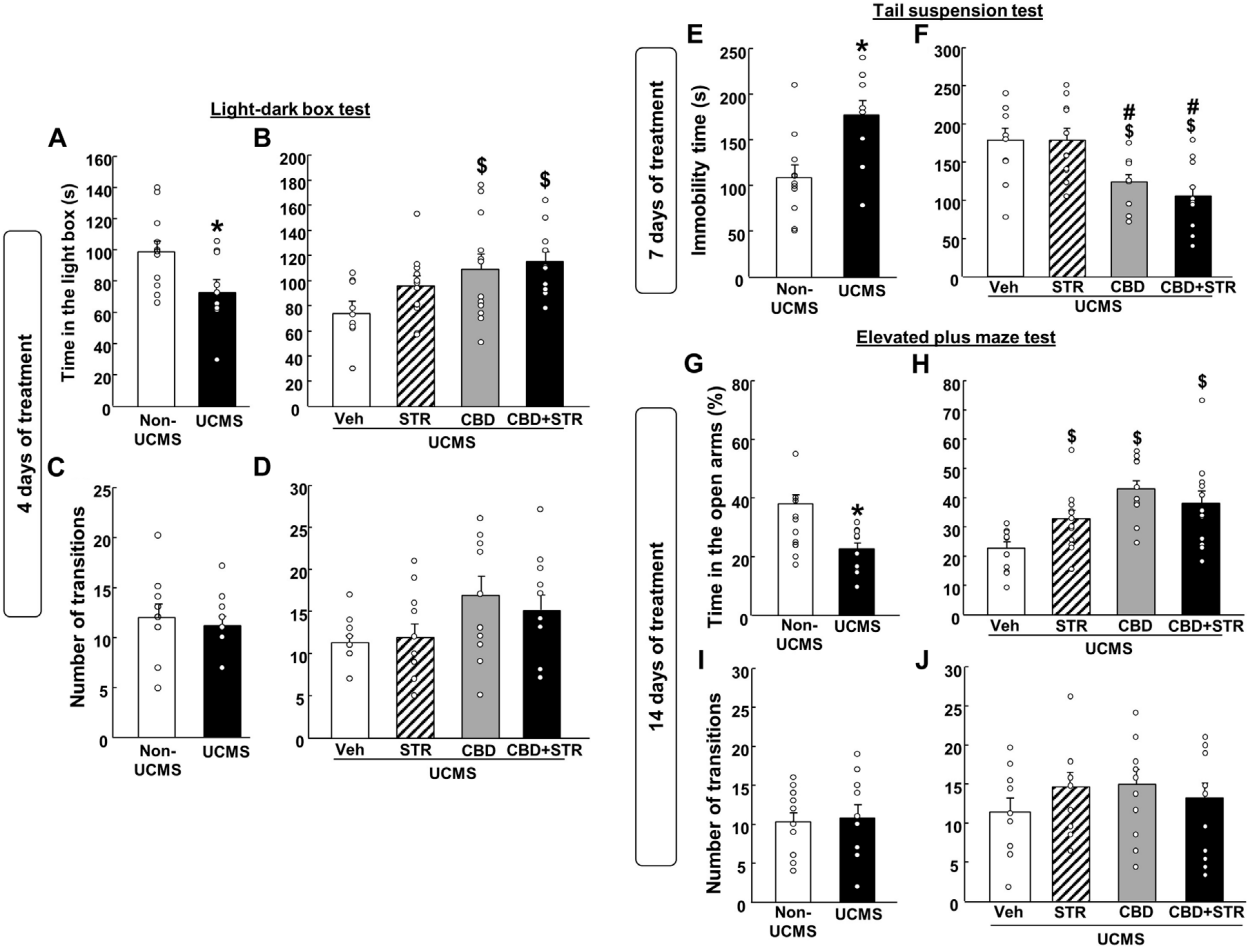
**Chronic effects of treatment with CBD, STR or their combination in modulating anxiety and behavioral despair in mice exposed to the unpredictable chronic mild stress model (UCMS).** Anxiolytic and antidepressant-like effects were evaluated at different time points (4, 7 and 14 days) using the light-dark box (panels **A–D**), tail suspension (panels **E** and **F**) and elevated plus maze (panels **G–J**) tests. Columns represent the means and vertical lines the ±SEM of each parameter evaluated. *Values from the VEH-treated UCMS group that differ significantly from VEH-treated non-UCMS group (Student’s t-test, *p* < 0.05). $ Values from STR-, CBD-, and/or CBD + STR-treated UCMS mice that were different from VEH-treated UCMS mice. # Values from CBD- and/or CBD + STR-treated UCMS mice that were different from STR-treated UCMS mice (One-way ANOVA followed by Student-Newman-Keuls, *p* < 0.05).

As expected, VEH-treated UCMS mice presented a reduced time spent in the lighted box compared with the VEH-treated non-UCMS mice (Figure 3A) (Student’s t-test: t = 2.452, 22df, *p* = 0.023) (n = 12/group). No differences were found in the number of transitions between both groups (Figure 3D) (Student’s t-test: t = 0.915, 22df, *p* = 0.371) (n = 12/group).

CBD treatment, alone or combined with STR, increased the time spent in the lighted box. Interestingly, no statistically significant differences were found between the STR-treated UCMS group and the vehicle-treated UCMS group (Figure 3C) (One-way ANOVA followed by Student-Newman- Keuls: F(3,44) = 3.753, *p* = 0.018) (n = 12/group). Regarding the number of transitions, there were no differences between the different groups (Figure 3D) (One-way ANOVA followed by Student-Newman-Keuls: F(3,44) = 2.469, *p* = 0.075) (*n* = 12/group).

#### 3.2.2 Effects at 7 days of treatment: TS test

Stressed mice treated with vehicle increased immobility time in the TS test compared to VEH-treated non-UCMS mice (Figure 3E) (Student’s t-test t = −3.209, 19df, *p* = 0.005) (n = 11 VEH-treated non UCMS; n = 12 VEH-treated UCMS).

Curiously, after 7 days of treatment, CBD significantly reduced the immobility time, alone or in combination with STR. In contrast, STR failed to induce any effect in comparison to its corresponding control group, UCMS treated with VEH (Figure 3F) (One-way ANOVA followed by Student-Newman-Keuls: F(3,40) = 7.991, *p* < 0.001) (n = 12/group).

#### 3.2.3 Effects at 14 days of treatment: EPM test

Mice exposed to the UCMS and treated with VEH increased the time spent (%) in open arms compared to VEH non-UCMS mice (Figure 3G) (Student’s t-test t = 3.970, 22df, *p* < 0.001) (n = 12/group). No differences were found in the number of transitions between both groups (Figure 3I) (Student’s t-test t = −0.213, 22df, *p* = 0.833) (*n* = 12/group).

Notably, the three pharmacological treatments significantly increased the time spent (%) in the open arms (Figure 3H) (One-way ANOVA followed by Student-Newman-Keuls: F(3,44) = 7.373, *p* < 0.001) (n = 12/group). No differences were identified in the number of transitions between the different groups (Figure 3J) (One-way ANOVA followed by Student-Newman-Keuls: F(3,44) = 0.792, *p* = 0.505) (n = 12/group).

#### 3.2.4 Effects at 18 days of treatment: NOR test

**FIGURE 4:**
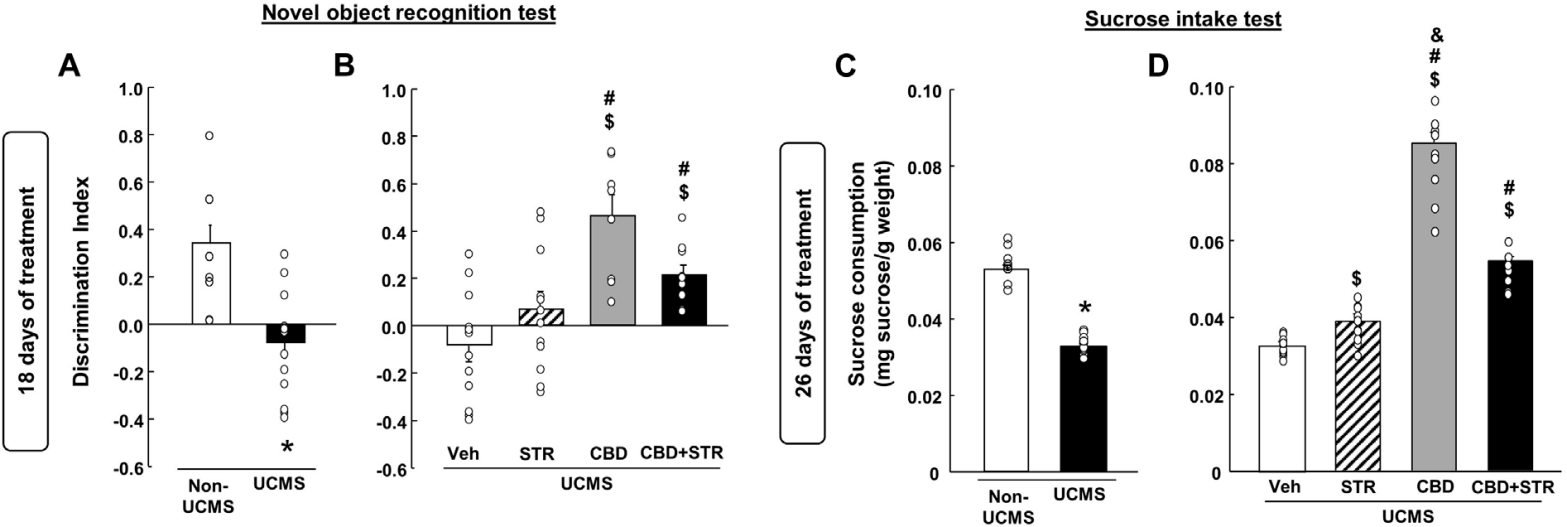
**Chronic effects of treatment with CBD, STR or their combination in modulating cognitive impairment and anhedonia in mice exposed to the unpredictable chronic mild stress model (UCMS)**. Panels **(A, B)** Novel Object Recognition Test (NOR) and panels **(C, D)** sucrose intake. Columns represent the means and vertical lines the ±SEM of each parameter evaluated. * Values from the VEH-treated UCMS group that differ significantly from VEH-treated non-UCMS group (Student’s t-test, *p* < 0.05). $ Values from STR-, CBD- and/or CBD + STR-treated UCMS mice that were different from the VEH-treated UCMS group. # Values from CBD- and/or CBD + STR-treated UCMS mice that were different from STR-treated UCMS mice. and Values from CBD-treated UCMS mice that were different from CBD + STR treated UCMS mice (One-way ANOVA followed by Student-Newman-Keuls, *p* < 0.05).

As shown in Figure 4A, VEH-treated UCMS mice presented a significant reduction of the discrimination index compared with its corresponding control group, VEH-treated non-UCMS (Student’s t-test, t = 4.179, 22df, *p* < 0.001) (n = 12/group).

The statistical analysis revealed that despite STR increased the discrimination index, this change did not reach statistical significance compared to VEH-treated UCMS mice. The treatment with CBD restored this alteration since CBD- and CBD + STR-treated UCMS mice showed a significant increase in the discrimination index compared to VEH- and STR-treated UCMS groups (Figure 4B) (One-way ANOVA followed by Student-Newman-Keuls: F(3,44) = 9.783, *p* < 0.001) (n = 12/group).

#### 3.2.5 Effects at 26 days of treatment: SI test

Mice exposed to the UCMS and treated with VEH showed a significant reduction in sucrose consumption compared to the VEH-treated non-UCMS group (Figure 4C) (Student’s t-test, t = 16.183, 22df, *p* = 0.001) (n = 12/group). STR, CBD and their combination significantly increased sucrose intake. Nonetheless, CBD was the drug that induced the most significant increase, being statistically significant compared to the groups treated with STR and CBD + STR (Figure 4D) (One-way ANOVA followed by Student-Newman-Keuls: F(3,44) = 212.745, *p* < 0.001) (n = 12/group).

### 3.3 Effects of chronic CBD, STR and its combination administration on modulating neuromolecular alterations induced by the UCMS

#### 3.3.1 Gene expression analyses by Rt-PCR

##### 3.3.1.1 Scl6a4, 5-HT1A and 5-HT2A in the DR, Hipp and AMY

**FIGURE 5:**
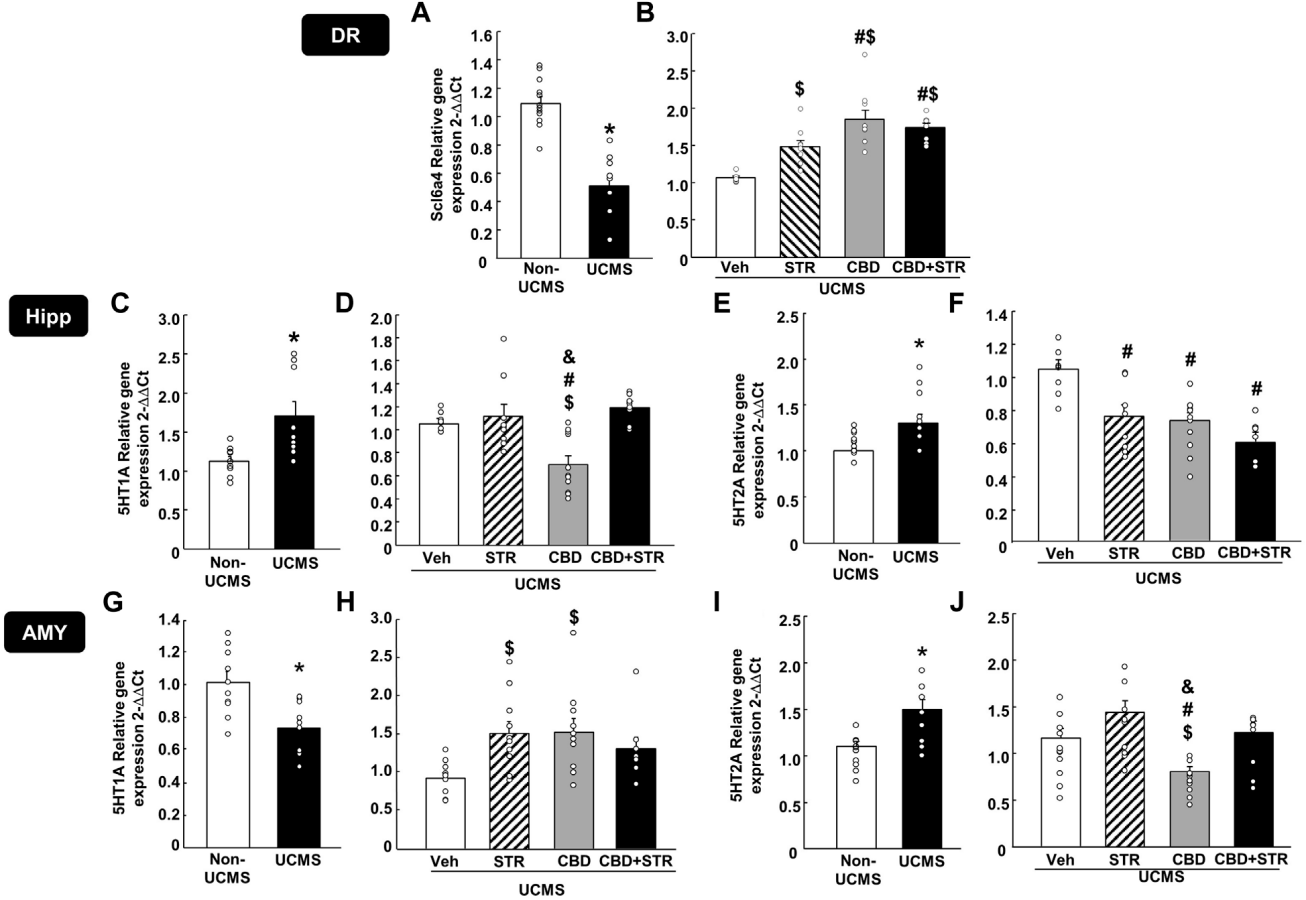
**Relative gene expression of the serotonin transporter (Slc6a4) and receptors (5-HT1A and 5-HT2A) in the dorsal raphe (DR), DG of the hippocampus (DG) and AMY (amygdala) of mice exposed to the UCMS and treated with CBD, STR or their combination by real-time PCR**. Columns represent the means and vertical lines the ±SEM of the Slc6a4 in the DR (panels **A, B**), 5-HT1A and 5-HT2A in the Hipp (panels **C–F**, respectively) and the AMY (panels **G–J**, respectively). *Values from the VEH-treated UCMS group that differ significantly from VEH-treated non-UCMS group (Student’s t-test, *p* < 0.05). $ Values from STR-, CBD- and/or CBD + STR-treated UCMS mice that were different from VEH-treated UCMS mice. # Values from CBD- and/or CBD + STR-treated UCMS mice that were different from STR-treated UCMS group, and Values from CBD-treated UCMS mice that were different from CBD + STR treated UCMS mice (One-way ANOVA followed by Student-Newman-Keuls, *p* < 0.05).

The results revealed that UCMS significantly reduced Scl6a4 gene expression levels in the DR (Figure 5A, Student’s t-test, t = 6.876, 19df, *p* < 0.001) (n = 11/group). Interestingly, STR, CBD and their combination significantly reversed that reduction. Notably, the increase induced by CBD, alone or in combination with STR, was more significant than that caused by STR alone (Figure 5B) (One-way ANOVA followed by Student-Newman-Keuls: F(3,40) = 15.626, *p* < 0.001) (n = 11/group).

In the Hipp, UCMS induced a significant increase of both 5-HT1A (Figure 5C) (Student’s t-test t = −2.923, 16df, *p* = 0.01) (n = 9/group) and 5-HT2A gene expressions (Figure 5E) (Student’s t-test t = −3.375, 18df, *p* = 0.003) (*n* = 11 VEH-treated non-UCMS; *n* = 9 VEH-treated UCMS). Among all treatments, only CBD reduced 5HT1A gene expression. No change was found in the UCMS groups treated with STR or with the CBD + STR combination (Figure 5D) (One-way ANOVA followed by Student-Newman-Keuls: F(3,32) = 9.554, *p* < 0.001) (n = 9/group). All the pharmacological interventions reduced 5-HT2A gene expression without any differences among them (Figure 5F) (One-way ANOVA followed by Student-Newman-Keuls: F(3,29) = 8.404, *p* < 0.001) (n = 9 VEH-treated UCMS; n = 8 STR-, CBD- and CBD + STR-treated UCMS groups).

In the AMY, UCMS induced opposite effects in the gene expression of both serotoninergic receptors since it reduced 5-HT1A (Figure 5G) (Student’s t-test t = 3.447, 18df, *p* = 0.003) (n = 10/group) but increased 5-HT2A gene expression (Figure 5I) (Student’s t-test t = −3.453, 18df, *p* = 0.003) (n = 10/group). Indeed, differences were also found between the different pharmacological approaches tested. On the one hand, CBD and STR increased 5-HT1A gene expression; however, in the case of the CBD + STR combination, no statistically significant change was found, although there was a tendency to reduce it (Figure 5H) (One-way ANOVA followed by Student-Newman-Keuls: F(3,34) = 3.861, *p* = 0.018) (n = 10 VEH-, STR- and CBD-treated UCMS groups; n = 8 CBD + STR treated UCMS). On the other hand, no change in 5-HT2A was found in the UCMS groups treated with STR or with the CBD + STR combination compared to VEH-treated UCMS mice. Notably, CBD was the only treatment reducing 5-HT2A gene expression, being this change statistically different from the other groups (Figure 5J) (One-way ANOVA followed by Student-Newman-Keuls: F(3,37) = 7.932, *p* < 0.001) (n = 11 VEH-treated UCMS; n = 10 STR-treated UCMS; n = 12 CBD-treated UCMS; n = 8 CBD + STR-treated UCMS).

##### 3.3.1.2 BDNF, PPARdelta and mVglut1 in the Hipp

**FIGURE 6:**
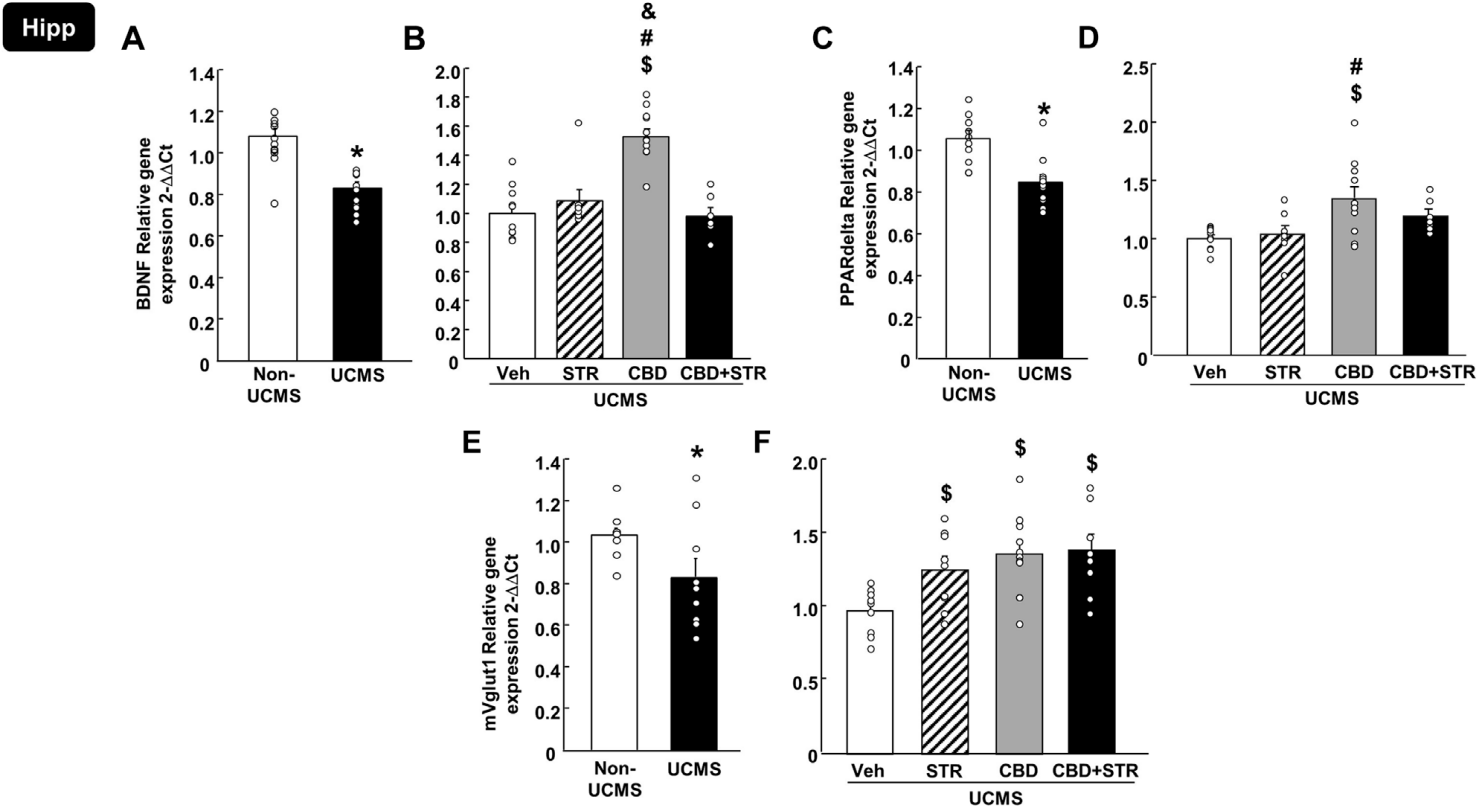
**Relative gene expression of brain-derived neurotrophic factor (BDNF), peroxisome proliferator-activated receptor delta (PPARdelta) and vesicular glutamate transporter 1 (mVglut1) in the hippocampus (Hipp) of mice exposed to the UCMS and treated with CBD, STR or their combination by real-time PCR**. Columns represent the means and vertical lines the ±SEM of the BDNF (panels **A, B**), PPARdelta (panels **C, D**) and mVglut1 (panels **E, F**). *Values from the VEH-treated UCMS group that differ significantly from VEH-treated non-UCMS group (Student’s t-test, *p* < 0.05). $ Values from STR-, CBD- and/or CBD + STR-treated UCMS mice that were different from VEH-treated UCMS mice. # Values from CBD- and/or CBD + STR-treated UCMS mice that were different from the STR-treated UCMS group. and Values from CBD-treated UCMS mice that were different from CBD + STR treated UCMS mice (One-way ANOVA followed by Student-Newman-Keuls, *p* < 0.05).

As shown in Figure 6A, relative gene expression of BDNF in the Hipp was reduced in mice exposed to the UCMS compared with the non-UCMS group (Figure 6A) (Student’s t-test, t = 5.281, 19df, *p* < 0.001) (n = 12 VEH-treated non-UCMS; n = 11 VEH-treated UCMS). Surprisingly, only CBD treatment reversed this alteration, increasing BDNF levels. No changes were induced by STR or the combination CBD + STR compared with the VEH-treated UCMS group (Figure 6B) (One-way ANOVA followed by Student-Newman-Keuls: F(3,38) = 19.676, *p* < 0.001) (n = 11 VEH-, STR- and CBD-treated UCMS groups; n = 9 CBD + STR UCMS).

Similarly, UCMS significantly reduced the relative gene expression of PPARdelta in the Hipp (Figure 6C) (Student’s t-test, t = 3.898, 19df, *p* < 0.001) (n = 12 VEH-treated non-UCMS; n = 11 VEH-treated UCMS). Notably, this alteration was reversed only by CBD treatment, with no effect induced by STR or CBD + STR combination. Besides, there was a statistical difference between CBD- and STR-treated UCMS mice (Figure 6D) (One-way ANOVA followed by Student-Newman-Keuls: F(3,34) = 5.288, *p* = 0.004) (n = 11 VEH- and CBD-treated UCMS groups; n = 8 CBD-treated UCMS and CBD + STR-treated UCMS).

Regarding mVglut1, there was a reduction in the UCMS group compared to VEH non-UCMS group (Figure 6E) (Student’s t-test, t = 2.203, 17df, *p* = 0.042) (n = 10/group). All pharmacological treatments reversed the effects induced by UCMS since they significantly increased mVglut1 gene expression. No differences were found among the different pharmacological approaches (Figure 6F) (One-way ANOVA followed by Student-Newman-Keuls: F(3,33) = 4.691, *p* = 0.008) (n = 10 VEH-treated UCMS and n = 9 STR-, CBD- and CBD + STR-treated groups).

#### 3.3.2 Conventional and confocal immunohistochemistry

##### 3.3.2.1 BDNF immunoreactive in the Hipp

**FIGURE 7:**
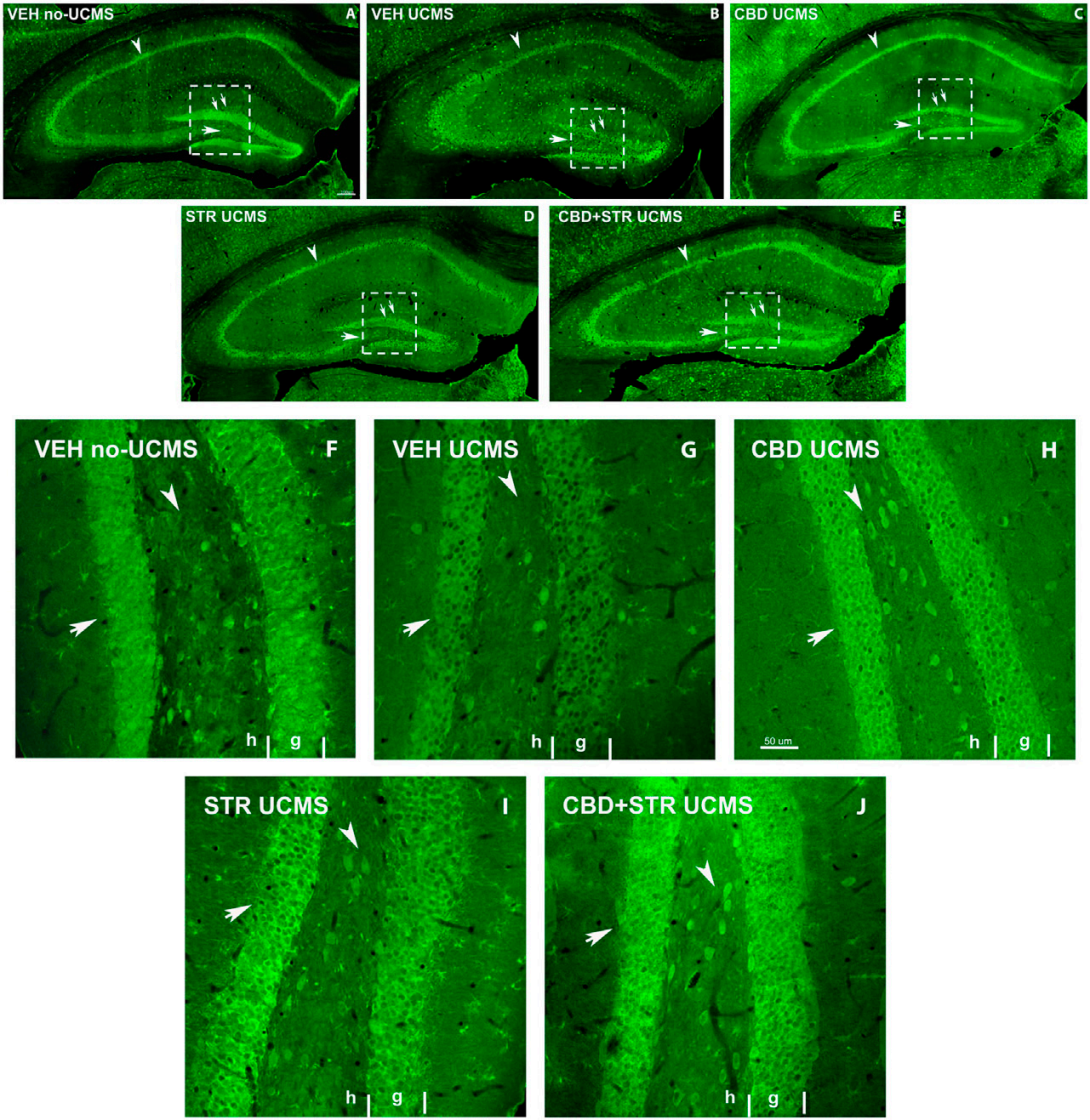
**Low magnification confocal images of BDNF-immunolabeling in mice exposed to the UCMS and treated with CBD, STR or their combination**. Collages of confocal photomicrographs showing BDNF-ir (green labeling; **A–E**) in the hippocampus of VEH-treated non-UCMS **(A)**, VEH-treated UCMS **(B)**, CBD-treated UCMS **(C)**, STR-treated UCMS **(D)** and CBD + STR treated UCMS **(E)** in adults’ mice. Note the decrease in the number and labeling of BDNF-in cells in the pyramidal layer of CA1 and CA3 (arrowhead), the granular layer (double arrow), and hilus (arrow) of DG in VEH-treated UCMS **(B)** compared with VEH-treated non UCMS **(A)**. These data show that BDNF-ir is recovered after treatment with CBD **(C)**, STR **(D)**, and CBD + STR **(E)**. The density and intensity of labeling are higher with CBD **(C)** and CBD + STR **(E)** treatment than with STR **(D)** alone in the pyramidal layer of CA1 and CA3 (arrowhead), the granular layer (double arrow), and hilus (arrow) of DG. High magnification of DG of mice exposed to the UCMS and treated with CBD, STR or their combination showed that the intensity of labeling is higher with CBD **(H)** and CBD + STR **(J)** treatments than with STR **(I)** (Arrows in **(F–J)**). G: granular; H: hilus.

BDNF immunostaining revealed a lower labeling intensity of the cells in all Hipp areas in the VEH-treated UCMS group compared with VEH-treated non-UCMS mice at low magnification (Figures 7A,B). On the other hand, a decrease of BDNF-ir cells in the hilus and granular layer of DG (double arrow Figures 7A–E), and the pyramidal layer of CA1 and CA3 was found in VEH-treated UCMS compared with VEH-treated non-UCMS. Interestingly, all the pharmacological approaches increased BDNF-ir compared with VEH-treated UCMS mice. In CBD- and CBD + STR-treated UCMS groups, the labeling intensity of BDNF-ir in pyramidal and granular cells was higher than in the STR-treated UCMS mice (Figures 7C–E). On the other hand, BDNF-ir in CA1 and CA3 decreased in VEH-treated UCMS compared with the VEH-treated non-UCMS group (Figures 7A,B). All the treatments increased BDNF-immunostained in both fields of Hipp (arrowhead in Figures 7C–E). Likewise, BDNF-ir in the hilus and the DG areas (arrow and double arrow Figures 7C–E) were decreased in the VEH-treated UCMS group (Figure 7B).

At high magnification, BDNF-ir in the hilus and the granular cells of DG in CBD-, STR- and CBD + STR UCMS groups increased compared with VEH-treated UCMS (Figures 7F–J). Even though BDNF-immunostained intensity was higher in STR-treated UCMS than in VEH-treated UCMS, the increase was more significant in the granular layer of DG in CBD and CBD + STR groups compared with STR-treated UCMS mice (arrow in Figures 7H–J). All these results revealed increased cell survival and plasticity in the UCMS mice treated with the different pharmacological approaches compared with VEH-treated UCMS mice. This increase was even more significant in mice treated with CBD and CBD + STR than with STR alone.

##### 3.3.2.2 NeuN and Caspase-3 immunoreactive in the Hipp

**FIGURE 8:**
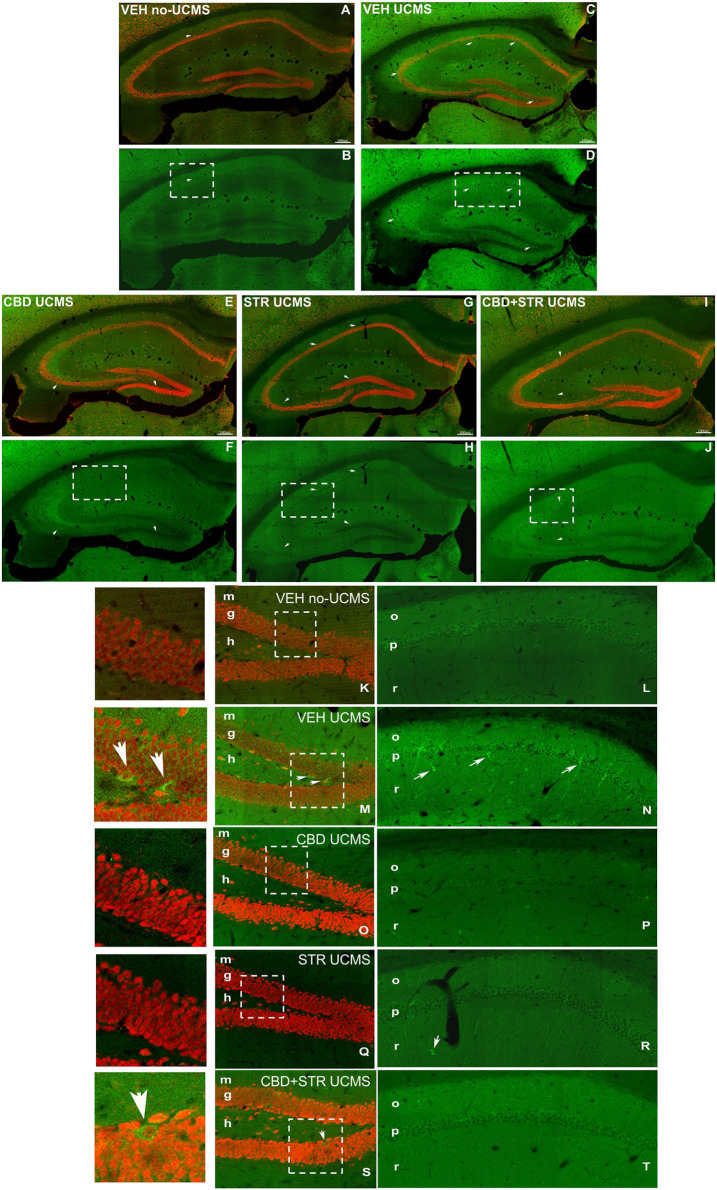
**Low magnification confocal image sowing NeuN/Caspase-3 double immunostaining coronal section of the hippocampus of mice exposed to the UCMS and treated with CBD, STR or their combination**. Low magnification photomicrographs of coronal sections of the hippocampus showing NeuN-ir neurons in red and Caspase-3-ir in green in VEH-treated non-UCMS **(A, B)**, VEH-treated UCMS **(C, D)**, CBD-treated UCMS **(E, F)**, STR-treated UCMS **(G, H)**, and CBD + STR treated UCMS **(I, J)**. Details of NeuN in DG **(K, M, O, Q, S)** and Caspase-3 **(L, N, P, R, T)** in CA1 of VEH-treated non-UCMS, VEH-treated UCMS, CBD-treated UCMS, STR-treated UCMS, and CBD + STR treated UCMS mice. Note the decrease of Caspase-3-ir in the granular layer of DG and pyramidal and stratum radiatum of CA1 in CBD-, STR-, and CBD + STR treated UCMS groups compared with VEH-treated UCMS group. The VEH-treated UCMS **(C, D)** group showed an increase in Caspase-3-ir in CA1, CA3, and DG compared with the treated groups **(D–I)** and VEH-treated non-UCMS group **(A, B)**. The UCMS mice treated with CBD **(E, F, O, P)** or CBD plus STR **(I, J, S, T)** showed lower Caspase-3-ir than mice treated with STR **(G, H, K, R)**. Same scale for **(A–J)** and for **(S, L, M, N, O, P, Q, R, S)**. High magnification of the CA1 showing the Caspase-3 positive cells in green in non-UCMS, UCMS, and treated with CBD, STR, or their combination **(L, N, P, R, T)** and NeuN/Caspase-3 double immunostaining (Caspase-3-ir in green and NeuN-ir in red, in the DG of the hippocampus **(K, M, O, S)**. Note that CBD-treated UCMS, STR-treated UCMS, and CBD+STR treated UCMS groups have less Caspase -3-ir than the VEH-treated UCMS (see details on the right). m: molecular; g: granular; h: hilus; o: oriens; p: pyramidal; r: radiatum.

In VEH-treated UCMS mice, the results of NeuN-Caspase-3 double-immunostained sections at low magnification (Figure 8A, C, E, G, I) revealed a decrease in NeuN-ir in CA1, CA3 and the hilus and granular cells of DG (arrows in Figure 8A, C, E, G, I) compared with its corresponding control group, VEH-treated non-UCMS. Interestingly, CBD, STR and the combination CBD + STR increased NeuN-ir.

On the other hand, Caspase-3-ir increased in VEH-treated UCMS compared to VEH-treated non-UCMS mice (Figure 8B, D). In contrast, Caspase-3-ir was lower in CBD-, STR-, and CBD + STR-treated mice compared with the VEH-treated UCMS group (arrows in Figure 8D, F, H, J). Between treatments, Caspase-3-ir was lower in UCMS mice treated with CBD- or CBD + STR than in those treated with STR alone (arrows in Figure 8F, H, J). Details of the CA1 region showed a decrease in NeuN-ir pyramidal cells in CA1 and an increase of Caspase-3-ir in VEH-treated UCMS compared with non-UCMS. Both CBD and STR, as well as their combination, reversed these alterations (arrow in Figure 8A, C, E, G, I). These results revealed higher cellular death, as noted by the decrease in NeuN-ir and the increase of Caspase-3-ir in VEH-treated UCMS compared with the VEH-treated non-UCMS group. Notably, the number of apoptotic cells decreased in mice treated with CBD- or CBD + STR even more than in those treated only with STR (arrows in Figure 8F, H, I).

## 4 Discussion

The present study revealed that the antidepressant action of CBD is faster and more effective than the reference drug used, STR, in modulating the behavioral and molecular disturbances induced by the UCMS. Indeed, the combination of CBD plus STR showed a worse outcome than CBD alone. These assumptions are based on the following observations: 1) CBD reversed anxiety and behavioral despair of stressed mice at 4, 7 and 14 days of treatment, while STR required 14 days to modulate the anxiogenic-like behavior induced by UCMS, 2) CBD significantly improved cognitive impairment and anhedonia of UCMS mice more than STR, 3) CBD normalized all the gene expression disturbances induced by the UCMS in critical elements within the serotoninergic system (Scl6a4, 5-HT1A and 5-HT2A) in the DR, Hipp and AMY compared to STR, 4) CBD increased BDNF and PPARdelta gene expressions in the Hipp of UCMS mice, whereas STR was without effects, 5) CBD increased BDNF and reduced caspase-3 immunoreactivities in the Hipp more than STR, 6) the combination of CBD plus STR showed a similar efficacy than CBD alone in the LBD, TST and EPM, however, a worse outcome was found in the improvement of cognitive impairment and anhedonia, and 8) the combination of CBD plus STR did not reverse the alterations induced by the UCMS in 5-HT1A, BDNF and PPARdelta in the Hipp, and 5-HT1A and 5-HT2A in the AMY; and increased BDNF and caspase-3 immunoreactivities in the Hipp less than CBD alone.

This study evaluated the antidepressant-like effects of chronic CBD administration compared with STR and the combination of both drugs in mice previously exposed to the UCMS experimental paradigm (Papp et al., 1996; Willner, 1997). The selective reuptake inhibitor STR was selected based on the fact that it is the first-line treatment of major depressive disorder (Cipriani et al., 2010) and one of the most common antidepressant drugs for treating other psychiatric diseases such as obsessive-compulsive disorder (Fenske and Schwenk, 2009), panic disorder (Hobgood and Clayton, 2009), posttraumatic stress disorder (Buhmann and Andersen, 2017) and social anxiety disorder.

Current antidepressant treatments, such as STR, require several weeks to reduce depressive symptomatology in patients and rats exposed to the UCMS, as previously reported (Ramanathan et al., 2003; Machado-Vieira et al., 2010; Lu et al., 2019). The LBD and TS tests revealed that CBD normalized the anxiogenic- and depressogenic-like effects induced by UCMS after 4 and 7 days of treatment, respectively. In contrast, the administration of STR required 14 days to produce a significant antidepressant action. This is the first evidence of the faster antidepressant-like effect of CBD compared to a reference antidepressant as STR.

In addition, CBD restored the cognitive impairments induced by UCMS, whereas STR tended to improve the cognitive impairment without reaching statistical significance. This finding contradicts previous results showing that STR reversed cognitive impairments caused by the UCMS in rats in the Water Morris and step-down inhibitory avoidance (SDIA) tests (Luo et al., 2016). These discrepancies may be due to differences in the strain used (CD1 mice vs. Sprague-Dawley rats), the duration of the UCMS (4 or 6 weeks) before the beginning of the pharmacological treatment, the STR doses (10 mg/kg vs. 5 mg/kg), and/or the behavioral test used (NOR vs. Water Morris and SDIA).

The results found in this study provide new relevant information suggesting that chronic CBD administration reversed all the behavioral alterations (increased anxiety, behavioral despair, cognitive impairment and anhedonia) induced by UCMS earlier than STR. Thus, these results lay the groundwork for future studies to explore the antidepressant profile of CBD, which could provide a better clinical benefit by inducing the effects faster and more effectively than other antidepressants.

The mechanisms of action of CBD were explored by measuring changes in the gene and protein expression of key targets closely related to depression. In this respect, monoaminergic neurotransmitters, especially serotonin (5-HT), have been widely involved in the pathophysiology of depressive disorders (Cowen and Browning, 2015). Recent studies have suggested that dopamine receptors D2 and D3 are involved in the mechanism of action of CBD (Seeman, 2016; Stark et al., 2020). Despite inconclusive results, numerous studies identified reduced activity of serotonin pathways, disruption of the 5-HTT and serotonin imbalance in depressive patients (Newberg et al., 2005; Savitz and Drevets, 2013). In rodents exposed to the UCMS, 5-HT concentrations (Gamaro et al., 2003; Bekris et al., 2005; Chengfeng et al., 2014), 5-HTT density and gene expression were reduced in several brain regions (Grecksch et al., 1997; Jahng et al., 2007; Lee et al., 2007; Liang et al., 2015). In this study, 5-HTT (Slc6a4) gene expression was significantly reduced in the DR of mice exposed to the UCMS compared with non-UCMS. Several reports related the increase of 5-HTT produced by antidepressants with the reduction of depressive-like behaviors and anhedonia (Tang et al., 2013; Qiu et al., 2014). Interestingly, CBD and STR increased Scl6a4 gene expression, although the effect was more pronounced with CBD. This may explain, at least in part, why CBD displays higher efficacy than STR in controlling the behavioral alterations induced by the UCMS.

Fourteen serotonin receptor subtypes have been identified. Among them, the 5-HT1A receptor plays a crucial role in controlling the concentrations of 5-HT. This receptor is widely distributed in the brain, with postsynaptic receptors in the cortex, Hipp and AMY, and somatodendritic autoreceptors in the raphe nucleus (Hjorth et al., 2000). Several studies support the involvement of the 5-HT1A receptor in the regulation of anxiety and depression, and neuroplasticity (Gross et al., 2002; Savitz et al., 2009; Zhang et al., 2010; Garcia-Garcia et al., 2014; Albert et al., 2019). Alterations of 5-HT1A gene expression and binding were found in the brain of rodents exposed to chronic stress (Albert et al., 2014; Bravo et al., 2014; Puglisi-Allegra and Andolina, 2015; Carneiro-Nascimento et al., 2021; Hale et al., 2021). PET studies showed alterations of 5-HT1A binding in cortical and raphe regions of depressive patients (Meltzer et al., 2004; Drevets et al., 2007). In our study, UCMS increased 5-HT1A receptor gene expression in the Hipp and decreased in the AMY. These results are consistent with previous reports suggesting that stressful stimuli induced opposite changes in 5-HT1A gene expression depending on the brain region analyzed (Bravo et al., 2014; Viudez-Martinez et al., 2018). CBD reversed both alterations, while STR only increased 5-HT1A in AMY. Based on previous studies, it is possible to suggest that CBD behaves as an agonist of the 5-HT1A receptor, which is responsible for its antidepressant properties (Russo et al., 2005; Zanelati et al., 2010; Gomes et al., 2013; Marinho et al., 2015; Linge et al., 2016; Sartim et al., 2016). Mood stabilizers and antidepressants significantly modify 5-HT1A functional activity in several brain regions (Hjorth et al., 2000; Nugent et al., 2013). In general, the antidepressant effects of SSRIs are potentially mediated by 5-HT1A receptors. In the raphe nuclei, the increase of serotonin concentrations induced by the inhibition of 5-HTT stimulates the presynaptic somatodendritic 5-HT1A receptors. This action results in an inhibition of serotonin release, which consequently delays the onset of antidepressant action. However, in chronic treatment, desensitization of the presynaptic 5-HT1A receptors occurs, increasing 5-HT concentrations in the synapse with the subsequent activation of postsynaptic 5-HT1A receptors and the appearance of antidepressant actions (Dong et al., 1999; El Mansari et al., 2005). Thus, the agonism of the 5-HT1A receptor, in combination with the 5-HTT inhibition, has been proposed to contribute to a faster antidepressant action (Hughes et al., 2005; Montalbano et al., 2019; Albert et al., 2021). Recently, it has been observed that repeated CBD treatment increased 5-HT firing activity by desensitizing 5-HT1A autoreceptors (Blier and De Montigny, 1983; De Gregorio et al., 2019), and this mechanism was associated with relieving pain-induced anxiety-like behavior in a rat model of neuropathic pain (De Gregorio et al., 2019). Thus, it is possible to hypothesize that the most efficient actions of CBD compared with STR on 5-HTT gene expression in the DR and the normalization of 5-HT1A gene expression alterations in Hipp and AMY produced by UCMS may be due to the activation of the 5-HT1A receptor. Future studies, for example, by blocking the 5-HT1A receptor in mice exposed to the UCMS and treated with CBD, are required to determine the significance of this serotonin receptor in the antidepressant effects of CBD.

An additional serotonin receptor affected by stress and involved in multiple psychiatric disorders is the 5-HT2A. Activation of this receptor in the AMY has been closely related to generalized anxiety disorder (GAD) (Nikolaus et al., 2010; Baeken et al., 2021), borderline personality disorders (Soloff et al., 2007) and PTSD (Murnane, 2019). Besides, cortical 5-HT2A increase was reported in suicide victims (Underwood et al., 2018; Odagaki et al., 2021) and several brain areas of rodents exposed to UCMS (McKittrick et al., 1995; Ossowska et al., 2001). In line with these findings, UCMS significantly increased 5-HT2A in the Hipp and AMY. Chronic CBD administration reduced these alterations, while STR decreased 5-HT2A gene expression only in the Hipp. Previous reports indicated that the increased expression of 5-HT2A is associated with unmitigated stress, increasing the intensity and consequences of stress rather than the relief of stress consequences (Pandey et al., 2002). Thus, it is tempting to speculate that reducing 5-HT2A induced by CBD may contribute to a more significant efficacy in reversing the behavioral disturbances induced by UCMS compared to STR. However, there is no information on how CBD interacts with the 5-HT2A receptor. The fact that it reduces the expression of 5-HT2A would indicate that it acts more as an agonist of this receptor. This should be further explored due to the emerging interest in 5-HT2A agonists for treating anxiety and depression, such as the LSD-like psychedelic drugs that acting as 5-HT2A agonists display rapid antidepressant effects related to their neuroplastic actions (Corne and Mongeau, 2019; Carhart-Harris et al., 2021; Tariq, 2022).

Impairment in brain neuroplasticity is one of the main etiopathogenic mechanisms underlying depressive disorders. It is known that the antidepressants’ efficacy lies in their ability to increase neuroplasticity. BDNF is a crucial regulator of synaptic plasticity, neurite outgrowth and neuronal connections in the brain (McAllister et al., 1999; Mamounas et al., 2000; Park and Poo, 2013) and mediates the plastic changes induced by antidepressants (Nibuya et al., 1995; Bjorkholm and Monteggia, 2016; Castren and Antila, 2017). In animal stress models, BDNF is downregulated in the Hipp (Nibuya et al., 1995; Nibuya et al., 1996; First et al., 2013; Gong et al., 2017; Zhu et al., 2019). The involvement of hippocampal BDNF in antidepressant efficacy has been demonstrated in different previous studies (Shirayama et al., 2002; Adachi et al., 2008; Castren and Rantamaki, 2010; First et al., 2013; Bjorkholm and Monteggia, 2016). In this study, BDNF gene and protein expressions were significantly reduced in the Hipp of mice exposed to the UCMS.

Interestingly, CBD treatment increased BDNF gene expression in the Hipp, in contrast to STR. Moreover, immunohistological studies revealed that the treatment with CBD increased BDNF immunoreactivity in different fields of the Hipp more significantly than STR. Our results are in agreement with previous reports showing that CBD increased BDNF gene expression and synaptophysin in the PFC and Hipp of ICR mice exposed to the UCMS (Xu et al., 2019). Besides, acute administration of CBD showed antidepressant effects associated with increased synaptophysin and PSD95 in the mPFC and BDNF protein expression in mPFC and Hipp (Sales et al., 2019). In additional animal models of brain ischemia, CBD increased hippocampal BDNF protein levels, stimulated neurogenesis and promoted dendritic restructuring (Mori et al., 2017). Similarly, in an animal model of mania induced by D-amphetamine, CBD increased BDNF expression in the Hipp (Valvassori et al., 2011). Furthermore, CBD treatment increased NeuN-ir and decreased caspase-3-ir more significantly than STR. Therefore, CBD was more effective in reversing the UCMS-induced increase in apoptosis and neuronal loss than STR.

The analyses of PPARdelta gene expression in the DG of the Hipp provided different results about the significant enhancement of neuroplasticity by CBD. PPARdelta belongs to the PPAR nuclear receptor family with a widespread brain distribution (Girroir et al., 2008; Tyagi et al., 2011). Recently, this receptor that regulates 5-HTT in the Hipp (Liu et al., 2017) is essential in reducing depressive-like behaviors (Ji et al., 2015) induced by stress. Moreover, the activation of PPARdelta induces neuroprotection and reverses neurodegeneration in Alzheimer’s disease (Tong et al., 2016), Parkinson’s disease (Martin et al., 2013) and Huntington’s disease (Dickey et al., 2016). Notably, the treatment with CBD was the only one that reversed the reduction of PPARdelta gene expression in the Hipp induced by the UCMS.

Potential changes in the vesicular glutamate transporter 1, mVGlut1, were also analyzed. This glutamate transporter is located in the presynaptic glutamate-releasing neurons, transporting glutamate into presynaptic vesicles and promoting synaptic glutamate release (Trudeau and El Mestikawy, 2018). The control of glutamate levels is essential to protect the brain from excess synaptic glutamate and excitotoxicity, which can lead to cell death (Shigeri et al., 2004). Unbalance between inhibitory and excitatory neurotransmission has been proposed to be involved in different psychiatric conditions, including depressive disorders (Selten et al., 2018; Duman et al., 2019). Recently, the modulation of glutamate neurotransmission has been related to the antidepressant effects of ketamine and its effects on neuroplasticity (Aleksandrova et al., 2017; Silberbauer et al., 2020). In this study, UCMS reduced mVGlut1 gene expression, which may be related to changes in glutamate concentrations in the synaptic cleft, whereas CBD and STR increased the expression of this gene in the Hipp. It is remarkable to mention that CBD was the only drug that reversed all the molecular alterations induced by the UCMS.

Another aspect analyzed in the present study was if the combination of CBD and STR may show additive or synergistic potential. Combining different drugs is a standard procedure for treating depressive disorders to achieve a more significant effect than individual drug therapies. This strategy also prevents specific dose-related side effects. Unexpectedly, the combination of CBD plus STR resulted in less effective than CBD modulating behavioral and molecular disturbances induced by UCMS, and similar, in some respects, to STR. These results raise the question about the nature of the interaction between STR and CBD, which makes their combination less valuable than CBD alone. One possible explanation is a potential pharmacokinetic interaction between CBD and STR since both drugs are hepatically metabolized by the cytochrome P450 (CYP450) enzymes (Wang et al., 2001; Watanabe et al., 2007; Jiang et al., 2011). Recently, *in vivo* and *in vitro* studies pointed out that CBD significantly inhibits CYP2C19, decreasing the metabolism of STR and additional SSRIs (Jiang et al., 2013; Anderson et al., 2021). This would increase plasma STR concentration and serotonin concentrations, reducing tolerability secondary to activation. Thus, the combined use of CBD plus STR appears to increase the risk of concentration-related SSRI side effects (Jakubovski et al., 2016; Vaughn et al., 2021). A recent case report showed a pharmacokinetic interaction between CBD and STR in a patient treated with STR for depression and anxiety. CBD inhibited CYP2C19 increasing STR exposure, producing hyponatremia and subsequent cognitive dysfunction (Nanan et al., 2022). This fact may be critical since the behaviors related to an excess of serotonin are similar to symptoms of anxiety and depressive disorders (Jukic et al., 2018). Moreover, CBD and STR bind to plasma proteins (Shahlaei et al., 2015; Liu et al., 2022). Thus, it would be interesting to explore a potential interaction that may cause STR or CBD concentrations to change and explains, at least in part, why the combination of both drugs has less efficacy than the administration of CBD alone.

On the other hand, time course and differences in the molecular neuroadaptations induced by the combination in comparison to CBD alone suggest some interaction between CBD and STR at the pharmacodynamic level. At the beginning of the treatment (1–14 days), CBD plus STR showed the same efficacy as CBD alone and less with STR. Notably, the time point (14 days) at which the effectiveness of the combination starts to be less effective than CBD is the same as when STR presents efficacy by itself. As treatment becomes chronic, the combination results in significantly less effectiveness than CBD, as shown by the results at 18 and 26 days for NOR and SC tests. Similarly, molecular neuroadaptation analyses showed that 5-HT1A, BDNF and PPARdelta are the main critical targets in which CBD differs from the combination and STR. CBD restored changes in 5-HT1A receptor gene expression in the Hipp, whereas neither the combination nor STR modified this target. CBD and STR separately changed the effects of UCMS on 5-HT1A receptor gene expression in the Amy, but not the combination. Notably, BDNF and PPARdelta gene expressions increased with CBD but not with STR or the combination. Altogether, these results suggest that the lack of effect modulating 5-HT1A, BDNF and PPARdelta in the Hipp is similar between STR and the combination. Despite further studies being necessary to determine the mechanisms underlying these observations, it is tempting to hypothesize that CBD and STR interact negatively in the modulation of the serotonergic system and neuroplasticity, reducing the efficacy of CBD when both drugs are administered together.

Taken together, the results of this study revealed that CBD induced an antidepressant-like effect in the UCMS accompanied by molecular neuroadaptations in crucial targets of the serotonin system and neuroplasticity, expanding the knowledge about its mechanism of action. CBD presents advantages over the antidepressant STR, notably significant speed and efficiency, to modulate behavioral despair, cognitive impairment and anhedonia, and all the molecular disturbances analyzed. More importantly, special attention should be given to the combination of CBD with current antidepressants since it appears to produce a negative impact on treatment.

In summary, this study lays the groundwork for future clinical studies to determine CBD’s efficacy and therapeutic positioning in treating depressive and anxiety disorders in humans.

## Data Availability

The original contributions presented in the study are included in the article/Supplementary Material, further inquiries can be directed to the corresponding author.
