# Supplementary material for: Unveiling behavioral and molecular neuroadaptations related to the antidepressant action of cannabidiol in the unpredictable chronic mild stress model
Source: Front Pharmacol. 2023 Apr 18;14:1171646. doi: 10.3389/fphar.2023.1171646 (PMC10151764; doi:10.3389/fphar.2023.1171646)
Supplement: Supplementary file 1 [file DataSheet1.pdf]

Supplementary Material

Unveiling behavioral and molecular neuroadaptations related to the antidepressant action of cannabidiol in the unpredictable chronic mild stress model

María Salud García-Gutiérrez, Daniela Navarro, Amaya Austrich-Olivares and Jorge Manzanares*

*** Correspondence:**
Jorge Manzanares: jmanzanares@umh.es

1 Dose-response effects of cannabidiol in mice exposed to the unpredictable chronic mild stress model

Briefly, CD-1 male mice were exposed to the UCMS protocol for 4-weeks. Once the UCMS model was established, mice will be divided into the following groups: VEH-treated UCMS (n=9), CBD 5 mg/kg-treated UCMS (n= 10), CBD 10mg/kg treated UCMS (n=10) and CBD 20mg/kg treated UCMS (n=9). CBD was dissolved as previously described and administered at the corresponding doses twice daily ip. Non-UCMS group was treated with the corresponding vehicle of CBD twice daily ip (VEH-treated non-UCMS, n=10).

CBD efficacy modulating behavioral alterations induced by UCMS were evaluated at different time points in the light-dark box (7 days), elevated plus maze (12 days), and tail suspension test (21 days).

In the LBD, the results revealed that VEH-treated UCMS mice spent less time in the light compartment compared with the VEH-treated non-UCMS group (figure S1A) (Student t-test: t=5.400, 17df, P<0,001). No differences were observed in the number of transitions (figure S1B) (Student t-test t=1.676, P=0.012). CBD, at the three doses tested, significantly increased the time spent in the light compartment in comparison with the VEH-treated UCMS group (figure S1C) (One way ANOVA followed by Student-Newman-Keuls F(3,37)=6.679, P=0.001). No differences were observed in the number of transitions between groups (figure S1D) (One-way ANOVA followed by Student-Newman-Keuls F(3,37)=0.822, P=0.49).

In the EPM, VEH-treated UCMS spent less time in the open arms compared with VEH-treated non-UCMS mice (Figure 1SE) (Student t-test t=4.426, P<0,001). No differences were observed in the number of transitions (figure S1F) (Student t-test t=-2.362, P=0.207). CBD significantly increased the time spent in the open arms at all the doses tested (Figure 1SG) (One-way ANOVA followed by Student-Newman-Keuls F(3,37)=11.643, P<0.001). No differences were observed in the number of transitions between groups (figure S1H) (One-way ANOVA followed by Student-Newman-Keuls F(3,37)=1.398, P=0.260).

As expected, in the TS test VEH-treated UCMS group showed an increased time of immobility compared with the VEH-treated non-UCMS (figure 1SI) (Student t-test: t= -9.199, 17 df, P<0,001). Curiously, only the high dose of CBD was able to reduce the immobility time in the TS test (figure 1SJ) (One-way ANOVA followed by Student-Newman-Keuls: F(3,37)=15,212, P<0,001.

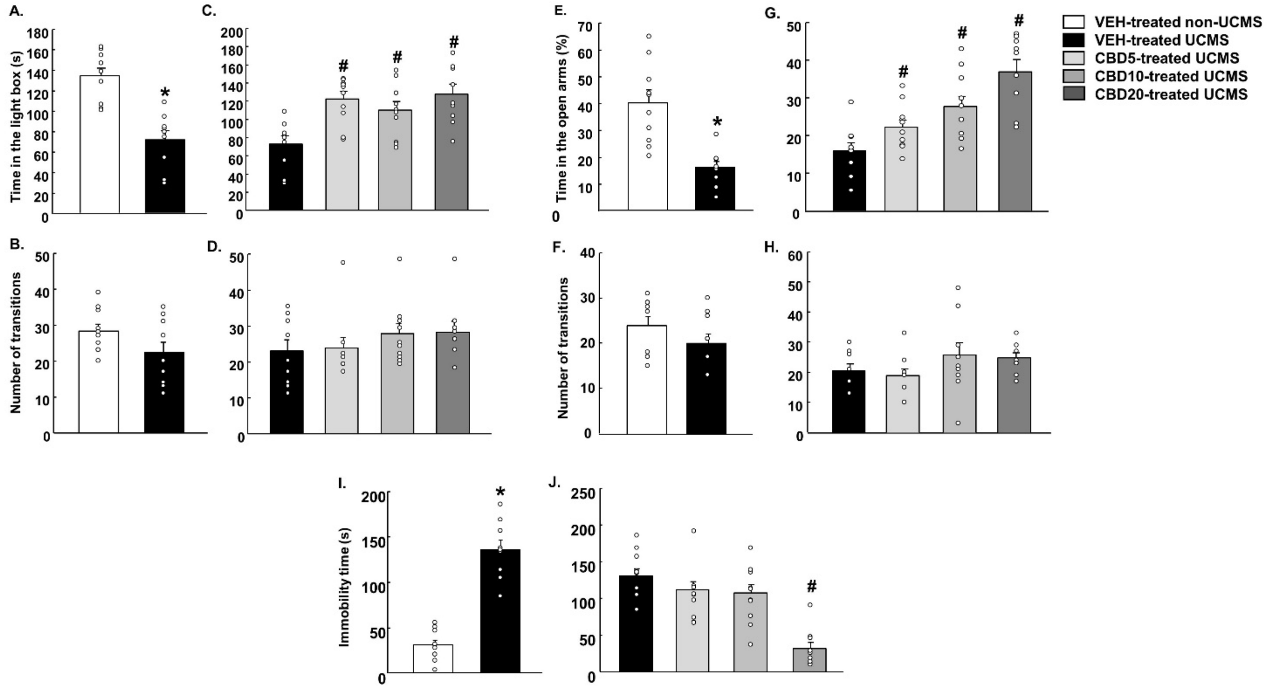

Figure S1. Dose-response effects of cannabidiol in mice exposed to the unpredictable chronic mild stress model. CD-1 male mice were exposed to the UCMS protocol for 4-weeks. Once the UCMS model was established, mice will be divided into the following groups: VEH-treated UCMS (n=9), CBD 5 mg/kg-treated UCMS (CBD5) (n=10), CBD 10mg/kg treated UCMS (CBD10) (n=10) and CBD 20mg/kg treated UCMS (CBD20) (n=9). CBD's effects on modulating behavioral alterations induced by the UCMS were evaluated at different time points in the light-dark box (panels A-D) (7 days), elevated plus maze (panels E-H) (12 days) and tail suspension test (panel I-J) (21 days). * VEH-treated UCMS group that differs significantly from the VEH-treated non-UCMS group. # treated-UCMS groups that differ from VEH-treated UCMS group.

frontiers
